# Intraoperative radiotherapy using a mobile electron LINAC: A retroperitoneal sarcoma case

**DOI:** 10.1120/jacmp.v6i3.2109

**Published:** 2005-08-17

**Authors:** A. Sam Beddar, Sunil Krishnan

**Affiliations:** ^1^ The University of Texas M. D. Anderson Cancer Center Departments of Radiation Physics; ^2^ Departments of Radiation Physics and Radiation Oncology Houston Texas U.S.A.

**Keywords:** intraoperative radiation therapy, mobile linear accelerator, Mobetron, Novac7, IORT

## Abstract

The advent of mobile LINACs for use in intraoperative radiation therapy (IORT) promises to make IORT more accessible than before and easier to deliver to patients undergoing surgery. Although mobile IORT systems have been available since 1999, few treatment centers currently use them. Here, we present the case of a typical patient undergoing IORT for retroperitoneal sarcoma to show how easy these mobile systems are to use and how adaptable they are within the operating room (OR) environment. We also discuss the roles and coordination of multidisciplinary team members during IORT and the feasibility of using mobile LINACs for IORT.

PACS number(s):

## I. INTRODUCTION

Intraoperative radiation therapy (IORT) offers the advantage of delivering a single high dose of radiation to a tumor or tumor bed after surgical resection and/or surgical exposure of high‐risk areas. The effectiveness of this treatment modality is significantly increased because the high doses of radiation are delivered to the target tumor or tumor bed while keeping the normal structures out of the radiation field. More than 20 years of clinical studies^(^ ^1^ ^)^ have shown IORT to be successful in treating a range of cancers, including primary T4 and recurrent rectal cancer,^(^ ^2^ ^–^ ^5^ ^)^ retroperitoneal sarcoma,^(^ ^6^ ^,^ ^7^ ^)^ pancreatic cancer,^(^ ^8^ ^–^ ^10^ ^)^ and some recurrent gynecological^(^ ^11^ ^–^ ^13^ ^)^ and genitourinary malignancies.^(^ ^14^ ^–^ ^17^ ^)^

The advent of the mobile LINAC has made IORT more accessible than before and easier to deliver to patients undergoing surgery for a number of reasons. One is that IORT can be used in any type of operating room (OR) because it requires no additional shielding.^(^ ^18^ ^,^ ^19^ ^)^ Another reason is that patients do not have to be transported to a radiation oncology department for treatment, so wound infections and problems associated with moving anesthetized patients are avoided. These difficulties have deterred many treatment centers from pursuing IORT over the last decade. For instance, Coia and Hanks^(^ ^20^ ^)^ reported in a patterns‐of‐care study^(^ ^21^ ^)^ that of the 1293 radiation oncology facilities in the United States in 1990, only 108 performed IORT. Today, fewer than 30 centers perform IORT. However, the availability of mobile IORT systems promises to bring about a significant revival of IORT and will make this special procedure accessible to a variety of clinical radiotherapy settings.

As an example, mobile systems have an advantage in the treatment of breast cancer in that they can be moved from the main OR (i.e., in‐patient surgery floor) to an out‐patient OR, where many hospitals now have their breast clinics. Recent studies have already shown the success of IORT^(^ ^22^ ^–^ ^24^ ^)^ in partial breast irradiation, an emerging treatment alternative for cancer. The addition of mobile systems to traditional IORT systems may advance the use of IORT for breast cancer patients and will certainly make this modality available to more of these patients.

**Figure 1:**
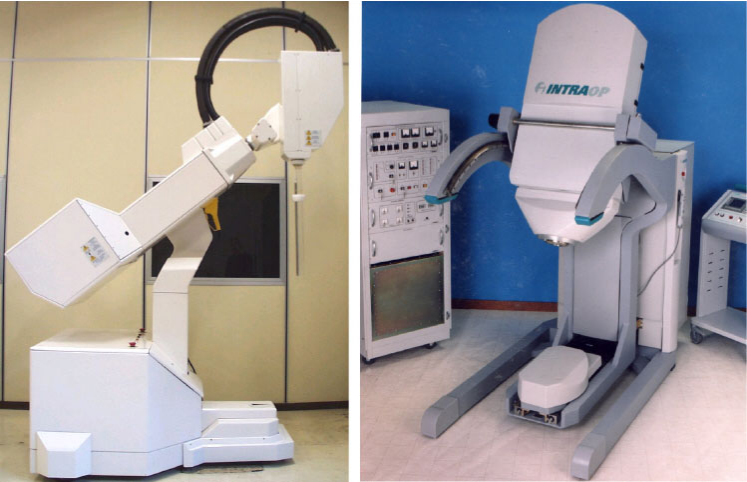
The Novac7, manufactured by Hitesys (left); the Mobetron, manufactured by IntraOp Medical Incorporated (right)

Currently, there are only two mobile IORT systems in use: the Novac7 (Hitesys, Milan, Italy), which is more widely accepted in Europe than elsewhere, and the Mobetron (Intraop Medical Incorporated, Santa Clara, CA), which is more established in the United States than in other countries (Fig. 1). Table 1 lists the 15 sites using the mobile Novac7 IORT system as of December 2004, and Table 2 lists the 10 sites using the Mobetron system.

**Table 1:** Sites using the Novac7 mobile IORT system

Country	Institution
Italy	Azienda Complesso San Filippo Neri, Rome
Italy	Azienda Ospedaliera Cà Foncello, Treviso
Italy	Azienda Ospedaliera Bianchi Melecrino, Reggio Calabria
Italy	IEO Istituto Europeo di Oncologia, Milan
Italy	EFO Istituto Regina Elena, Rome
Italy	Azienda Ospedaliera Renzetti, Lanciano
Italy	Ospedale della Misericorida, Grosseto
Italy	Ospedale di Cisanello, Pisa
Italy	Villa Flaminia, Private Hospital, Rome
Italy	Cancer Institute, Bari
Italy	A. Businco Cancel Institute, Cagliari
Italy	San Vincenzo Hospital. Taormina
Italy	Alta Valle del Tevere Hospital, Città di Castello
Greece	Ospedale Saint Savvas, Athens
Germany	Universitätsklinikum, Aachen

**Table 2:** Sites using the Mobetron mobile IORT system, plus the year of installation

Country	Institution	Year of installation
United States	University of California San Francisco, California	1997
United States	University Hospitals of Cleveland, Ohio	1999
United States	University of Louisville, Kentucky	2000
United States	University of North Carolina, Chapel Hill, North Carolina	2001
United States	Mayo Clinic, Scottsdale, Arizona	2002
United States	Methodist Hospital of Indianapolis, Indiana	2002
the Netherlands	Catharina‐ziekenhuis, Eindhoven	2003
Poland	University Hospital, Krakow	2003
Spain	Hospital San Jaime, Torrevieja	2004
United States	Ohio State University Hospital, Columbus, Ohio	2004
Italy	Ospedale Maggiore della Carita, Novara	2005

Both systems produce high‐energy electron beams, with the main difference between the two being their gantry docking systems. The Novac7 uses a hard‐docking system in which the gantry is in direct physical contact with the electron cone applicator, a cylindrical collimating cone that directs the electron beam to the treatment area. The Mobetron, on the other hand, uses a soft‐docking system in which the gantry is optically guided to a position 4 cm above the applicator.

Although mobile IORT systems have been on the market since 1999, they have not yet been broadly accepted. For example, only seven centers in the United States have adopted the Mobetron system. This underuse would seem to imply that the many advantages these systems offer are simply unknown to the medical community. For this reason and because of our success with the Mobetron system, we wanted to explain the application of this underutilized technology and show its feasibility in clinical settings. We have described in detail a typical IORT case using the Mobetron and the roles and coordination of the multidisciplinary IORT team members during the procedure.

### A. Description of the Mobetron and electron beam characteristics

**Figure 2:**
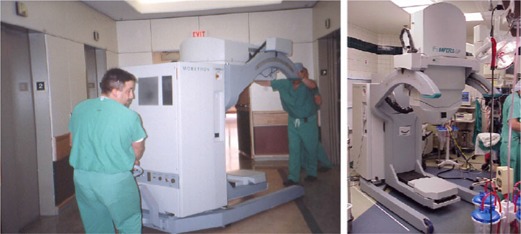
The Mobetron therapy unit en route to the OR (left) and ready for treatment (right)

The Mobetron system is composed of three separate units: the control console, the modulator, and the therapy module.^(^ ^18^ ^,^ ^25^ ^)^ The control console, which operates the accelerator during radiation treatment delivery, is placed outside the OR so that the radiation treatment delivery is controlled remotely. The modulator houses the electronic system of the accelerator and energizes the accelerator to produce the electron beams. The therapy module (Fig. 2) houses the accelerator guide and control systems that generate and deliver the radiation.^(^ ^26^ ^)^ The Mobetron system produces four levels of energy—4 MeV, 6 MeV, 9 MeV, and 12 MeV—with therapeutic ranges up to 4 cm (Table 3). The system is designed to deliver a very large, uniform dose of 10 Gy to 25 Gy in a single fraction at a dose rate of 10Gy/Gy.

**Table 3:** Typical electron beam characteristics produced by the Mobetron for the given range of energies

Electron energy (MeV)	Surface dose (%)	Therapeutic range^a^ (cm)	100% depth (cm)	5% depth (cm)	X‐ray contamination (%)
4	82	1.0	0.7	1.8	0.3
6	82	1.7	1.2	2.8	0.4
9	89	2.8	2.0	4.7	0.7
12	94	3.7	2.6	6.0	0.8

a The therapeutic range is defined as the depth that corresponds to the 90% level after the peak depth.

**Figure 3:**
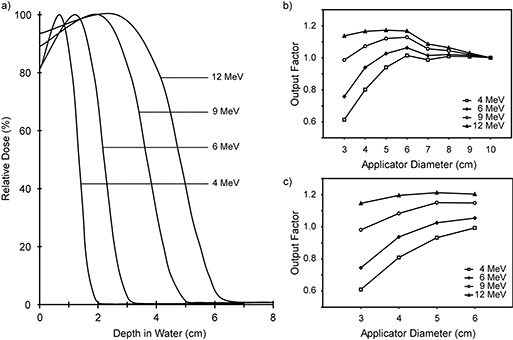
(a) Depth‐dose curves for the 4‐MeV, 6‐MeV, 9‐MeV, and 12‐MeV electron beams for the 10‐cm diameter applicator. The X‐ray contamination corresponding to each energy is shown in Table 3. (b) Output factors for flat applicators relative to the 10‐cm diameter applicator. (c) Output factors for beveled applicators relative to the 10‐cm diameter flat applicator.

**Figure 4:**
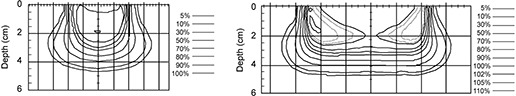
Isodose distributions for a 9‐MeV electron beam using a 4‐cm flat applicator (left) and a 10‐cm flat applicator (right) (Ref. 10)

**Figure 5:**
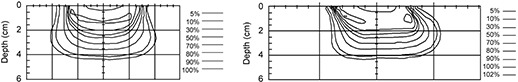
Isodose distributions for a 9‐MeV electron beam using a 6‐cm beveled applicator along both the transverse and longitudinal axes (Ref. 10)

The depth‐dose curves showing electron penetration as a function of distance from the surface are shown in (Fig. 3a). Treatments are delivered using either flat or beveled circular applicators. Flat applicators are used for sites where treatment areas are predominantly flat, and beveled applicators are used to treat areas that present themselves at an angle. Eight flat applicators in 1‐cm increments ranging from 3 cm to 10 cm in diameter and four beveled applicators (3 cm, 4 cm, 5 cm, and 6 cm diameter) are available. The output factors for the entire set of applicators relative to the 10‐cm flat applicator are shown in (Figs. 3b) and (c). Typical isodose distributions^(^ ^10^ ^)^ for selected flat and beveled applicators are shown in Figs. 4 and 5.

### B. Patient assessment and treatment planning

A 59‐year‐old woman presented with progressive abdominal bloating, early satiety, weight gain, and nonspecific right flank discomfort. Imaging of her abdomen revealed a large mass in the right upper quadrant of the retroperitoneum. She underwent primary resection at an outside facility, and the pathology report showed a well‐differentiated liposarcoma. The margins were negative. No adjuvant therapy was given. She was followed up routinely with clinical examinations and imaging studies and showed no evidence of disease for two years. Approximately two years after her initial treatment, she noted a firm area in her right flank. She complained of minimal right flank discomfort but had no other symptoms. Apart from her prior sarcoma resection, her past medical history was otherwise unremarkable.

A CT scan of her abdomen and pelvis showed a large recurrent fatty tumor in the right upper quadrant of the retroperitoneum. The tumor displaced the kidney inferiorly and the vena cava, duodenum, and pancreas medially. There was no evidence of liver metastases. No lung metastases were found with the chest CT. A CT‐guided core‐needle biopsy histologically confirmed the diagnosis of a recurrent liposarcoma.

After discussions among a multidisciplinary team including a surgical oncologist, a radiation oncologist, and a medical oncologist, it was decided to offer the patient preoperative radiation therapy followed by surgical resection and intra‐operative radiation therapy. A preoperative external beam radiation therapy (EBRT) dose of 45 Gy was delivered in 25 fractions over a five‐week period. Surgery was scheduled six weeks after completion of the EBRT.

### C. Perioperative phase

The perioperative phase of IORT involved patient assessment and other duties performed by the IORT nurse, equipment setup and quality assurance, and the surgical procedure.

#### C.1 Mobetron setup and quality assurance

The night before surgery, the modulator and therapy module of the Mobetron were moved to the OR. Quality assurance of the Mobetron was performed the morning of surgery. Door interlocks, preventing function of the Mobetron when activated, were checked for functionality, as were the mechanical motions and docking system of the unit. The dose‐rate output and energy of the accelerator were measured using standard procedures for all energies.^(^ ^27^ ^,^ ^28^ ^)^ Following completion of all quality assurance tests, the measured dose rate output was used to calculate the amount of radiation (monitor units) to be delivered to the patient after the total dose prescribed by the radiation oncologist.

The IORT nurse coordinated the IORT‐related procedures in the OR and assisted the circulating nurse as needed. She verified that the necessary sterile and nonsterile IORT supplies^(^ ^27^ ^)^ were readily available and began the tasks on the nursing IORT checklist.^(^ ^26^ ^)^ She measured the patient's height, along with the approximate location of the tumor with respect to that height. She helped the anesthesiology team plan patient positioning based on the patient's height and on the location of the tumor within the space of the surgical bed and its extension.

#### C.2 Surgery

At the beginning of the procedure, the surgeons were reminded that the radiation oncology team needed 30‐min's notice for the anticipated treatment delivery. The mass was resected, as was the right kidney. The tumor had compressed the vena cava and had extended under the liver. A gross total resection resulted in close but negative margins, which were identified as the areas to be treated with IORT. Clips were placed to mark the extent of the tumor.

### D. Intraoperative phase

The intraoperative phase of IORT involved setup, docking of the mobile unit, and treatment delivery.

#### D.1 Setup

**Figure 6:**
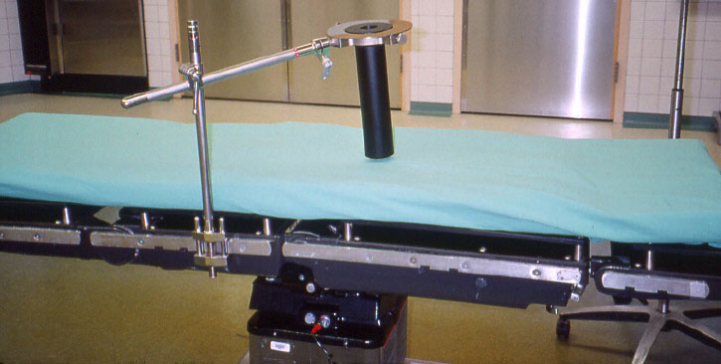
The modified Bookwalter surgical clamp stabilizing the electron cone applicator to the bed rail. The mirror clamp, used as a guide to the optical docking system, can be seen above the black applicator.

After surgery, a TV monitor was moved into the hallway. Two movable door panels were placed at the opposite ends of the hallway to limit traffic in this area and to help maintain sterility of the scrubbed team, whose members remained in the hall during the actual treatment delivery. When the radiation oncologist arrived, he donned a headlight and then scrubbed in. After viewing the tumor site and treatment area, he assessed the exact size of the treatment area and requested a 10‐cm flat cone applicator. He inserted a bolus (a Lexan disk) to decrease the depth of the electron beam's penetration and to direct more energy to the treatment surface. The insert was secured to the cone applicator with adhesive paper tape. The cone applicator was then inserted into the mirror clamp and directed to the treatment site. The applicator was stabilized by a modified Bookwalter surgical clamp (Codman & Shurtleff, Inc., Raynham, MA) (Fig. 6) attached to the rail of the OR bed. The site was verified by both the surgeon and the radiation oncologist.

**Figure 7:**
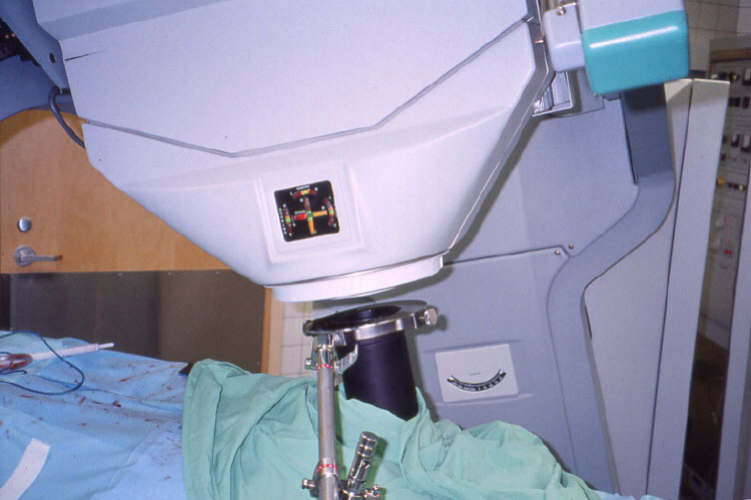
The Mobetron with the gantry soft‐docked to the electron cone applicator. Note the air gap between the end of the gantry and the applicator (4‐cm air gap).

The radiation oncologist then prescribed the treatment dosage based on the extent of the tumor, the patient's anatomy, the amount of residual disease, and the amount of prior radiation delivered to the site. The radiation therapist applied the sterile collimator cap to the treatment head of the Mobetron. The instrument tables were moved to a far corner of the room so that sterility could be maintained during the docking process. A sterile drape was placed around the cone applicator to maintain field sterility during docking.

#### D.2 Docking

The OR bed (with a sliding top to facilitate the docking procedure) was reversed, and a foot extension was placed at the head of the bed. This placement allowed enough room from the end of the bed to clear the Mobetron base and the beam stopper for docking.

The docking mechanism of the Mobetron unit relies on an optical docking system consisting of laser detecting devices mounted on the accelerator beam collimation system. These devices help the operator perform the soft‐docking of the gantry with the electron applicator (Fig. 7). This soft docking was achieved by adjusting the gantry rotation angle and tilt angle, height, and two translational shifts (longitudinal and lateral) in the horizontal plane. The status of each aspect of the alignment, shown on the LED light display (Fig. 7) on the accelerator, helped the operator guide the gantry during the docking process.

**Figure 8:**
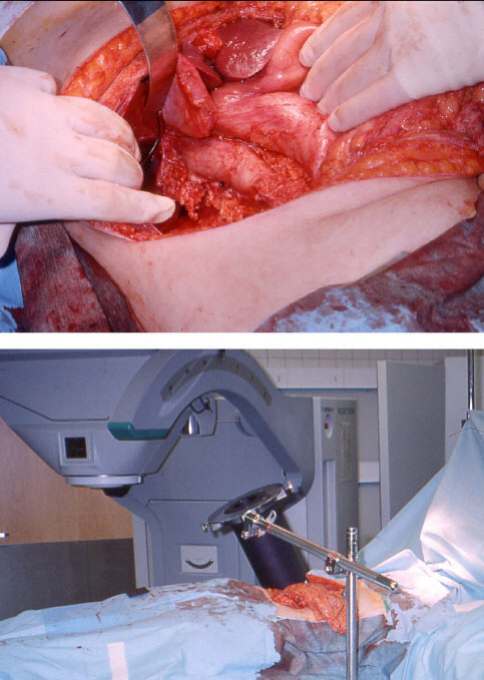
This case illustrates the effectiveness of IORT, in which high doses of radiation can be delivered to the target tumor or tumor bed without involving the normal structures.

All tubings and cords from the sterile field (suction, electrocautery, etc.) were disconnected for ease in moving the OR table. During the docking procedure, the anesthesiologist, nursing team, and surgical team together moved the OR table toward the Mobetron to precisely align the cone under the treatment head. The table was moved slowly and carefully to maintain sterility, the patient's position, and the location of the cone applicator to prevent any patient injury. The docking took 8 min, after which the entire team exited into the hallway. The anesthesiologist focused the TV camera on the patient's monitors and then connected it to the TV monitor in the hall. Doing so enabled him to monitor the patient's vital signs from the hall. The doors to the sterile core (on the opposite side of the room) were locked to prevent anyone from entering during treatment delivery.

#### D.3 Treatment delivery

For this patient, the dose prescribed by the radiation oncologist was 12.5 Gy (1250 cGy). The radiation oncologist requested treatment coverage extending from the surface to a depth of at least 0.5 cm to be encompassed by the 90% isodose line. After reviewing the dosimetry data, the medical physicist and the radiation oncologist determined that using a 6‐MeV electron beam, with a 0.5‐cm bolus, prescribed to the 90% isodose line would adequately cover the targeted volume. The 0.5‐cm bolus was used to increase the surface dose from 82% to 90% while still maintaining a good coverage at depth. For the beam defining field size, a 10‐cm circular flat cone applicator was selected to adequately cover the target volume with an appropriate margin around the tumor bed.

The medical physicist and radiation therapist programmed the control console. Their calculations were double‐checked and the console settings verified. The radiation treatment, lasting 2 min, was then delivered. After the treatment, the entire team reentered the OR.

The patient was then moved from under the Mobetron back to the center of the OR. The radiation oncologist viewed the location of the cone applicator to verify that the treatment had been delivered to the appropriate site. He then removed the cone applicator, mirror clamp, and Bookwalter surgical clamp system from the field and gave them to the IORT nurse, who took them to the decontamination room for cleaning, inspection, wrapping, and sterilization. The circulating nurse reconnected all tubings and cords in preparation for the conclusion of the surgical procedure.

#### D.4 Completion of surgery

The surgeons closed the incision, during which time the nurses counted the closing sharps, sponges, and instruments. The circulating nurse then called an operative report to the postanesthesia care unit. When the procedure was over, a dressing was applied. The electro‐cautery pad was removed, and the patient's skin condition was assessed. The patient was then transferred to the postanesthesia care unit.

The circulating nurse documented the IORT as part of the surgical procedure. She also documented the names of all the radiation oncology staff involved in the procedure.

The IORT nurse completed the nursing IORT checklist and placed it in the nursing notebook on the nonsterile IORT cart. The TV monitor, TV camera, and X‐ray shields were returned to their proper storage locations. The Mobetron components were cleaned and returned by the radiation therapists to the equipment storeroom.

#### D.5 After surgery

The patient did well postoperatively and was discharged on the fifth postoperative day. The pathology study revealed a 6‐cm sarcoma consistent with pleomorphic liposarcoma, which was different from the previously diagnosed well‐differentiated liposarcoma.

## II. DISCUSSION

Retroperitoneal sarcomas account for 10% to 20% of all soft tissue sarcomas. They differ from their counterparts that occur at other sites, such as the extremities, in that prognosis is poorer, with a five‐year survival of less than 50% and a relative lack of clinical relevance of tumor grade for local control. They pose a unique challenge for the treating oncologist because they are often large and involve adjacent critical normal structures. The mainstay of treatment is surgical resection. Gross total resection that results in negative margins is a known positive prognostic factor.^(^ ^29^ ^–^ ^33^ ^)^ However, anatomic limitations due to surrounding normal tissues often result in close or positive resection margins.^(^ ^33^ ^–^ ^37^ ^)^ Recurrences after resection are predominantly local, although distant metastases have also been noted. Despite optimal surgical resection, recurrences are noted in 70% to 90% of patients and continue to occur many years after surgery.^(^ ^35^ ^–^ ^37^ ^)^ The resectability rate for recurrent retroperitoneal sarcomas is similar to that for primary sarcomas, but the survival rate is believed to be less than half that for sarcomas that recur after the primary resection.^(^ ^38^ ^)^ More recent data suggest that recurrences present a renewed opportunity for curative treatment since recurrences are rarely associated with metastatic disease.^(^ ^39^ ^)^

The addition of radiation therapy to improve local control is an extrapolation of the benefit observed in extremity sarcomas. As with the challenges faced by surgical oncologists, radiation oncologists are limited by what can be done with routine EBRT techniques. The large volumes of bowel, kidney, and liver often within the treatment field limit the ability to give more than 45 Gy to 50 Gy of radiation. An elegant solution to this problem has been to administer an additional dose of radiation intraoperatively, when critical structures can be surgically and physically displaced away from the treatment field, as illustrated in Fig. 8.

EBRT can be administered preoperatively or postoperatively, and the dose is generally 45 Gy to 50 Gy (the dose limit for small bowel tolerance). We prefer preoperative radiation therapy for a number of reasons:

1. The tumor itself displaces abdominal organs, limiting their exposure to radiation and minimizing toxicity.
2. A dose of preoperative radiation may be more effective than an identical dose of postoperative radiation given that postoperative therapy involves treating a potentially hypoxic (less radiosensitive) tumor bed.
3. A preoperative dose may reduce the size of the tumor that needs to be resected and may make an unresectable tumor resectable.
4. Such a dose may reduce the likelihood of the tumor's seeding the retroperitoneum or abdomen during resection.
5. Defining the tumor area on the basis of CT scans is easier to do preoperatively than it is postoperatively.

The approach described above of IORT and preoperative or postoperative EBRT has been shown in numerous large groups to be feasible and to have acceptable levels of toxicity.^(^ ^40^ ^–^ ^44^ ^)^ In those groups, there seems to have been better local control than in groups treated without IORT. The only randomized study to have been performed under these conditions was conducted by the National Cancer Institute. Patients were randomized to high‐dose postoperative

EBRT (50 Gy to 55 Gy) versus IORT (20 Gy) and postoperative EBRT (35 Gy to 40 Gy) after marginal resection of retroperitoneal sarcomas.^(^ ^45^ ^)^ This study showed a longer time to recurrence and a lower frequency of in‐field recurrence when IORT was used but no survival advantage. In addition, the IORT patients had significantly less chronic toxicity.

Although the roles of EBRT and IORT have not been conclusively established by prospective randomized trials, it does seem reasonable to assume that radiation therapy confers a local control benefit with acceptable toxicity. When these two therapies are used together, a team of experienced surgical and radiation oncologists should coordinate the treatment. Adding intensity‐modulated radiation therapy to the EBRT component of treatment can help minimize morbidity by limiting the treatment dose to the bowel, kidneys, liver, and spinal cord.^(^ ^46^ ^,^ ^47^ ^)^ The dose‐limiting toxicity for IORT is neurotoxicity, and this seems to be greater when doses of 15 Gy or more are used.^(^ ^48^ ^,^ ^49^ ^)^ Late ureteral toxicity sometimes occurs when the ureter is in the high‐dose region.^(^ ^50^ ^)^ Abutment of two IORT fields increases the risk of toxicity in the overlap region. In addition to electron IORT, high‐dose rate IORT is also an option for delivering the localized boost dose of radiation intraoperatively.^(^ ^51^ ^)^

The role of chemotherapy in the treatment of retroperitoneal sarcomas is still investigational. Recent reports document acceptable toxicity with the combination of continuous‐infusion adriamycin and concurrent preoperative radiation therapy followed by resection and intraoperative radiation therapy.^(^ ^7^ ^)^ Collaborative efforts are needed for systematic, randomized trials of treatments for this disease. Advances in technology (e.g., mobile IORT and intensity‐modulated radiation therapy) should be exploited to improve the therapeutic ratio and to standardize the multimodality treatment of retroperitoneal sarcomas.

## III. CONCLUSION

We have presented a typical case in which a mobile LINAC was used for IORT, and we have demonstrated the relative logistical ease of such a procedure. It is our hope that, as the medical community becomes more familiar with the use of mobile LINACs, a greater number of treatment centers will adopt this still new technology, thus making IORT more accessible to patients in a variety of clinical settings.
